# Development and validation of a nomogram for peritoneal dialysis-associated peritonitis

**DOI:** 10.1080/0886022X.2026.2724570

**Published:** 2026-10-01

**Authors:** Zhiwu Tian, Shuang Zhou, Biwen Tian, Xiaoying Yang, Meifang Mai

**Affiliations:** Department of Nephrology, The Fifth Affiliated Hospital of Sun Yat-sen University, Zhuhai, Guangdong, China

**Keywords:** Peritoneal dialysis, Peritoneal dialysis-associated peritonitis, nomogram, prediction model, survival analysis

## Abstract

Peritoneal dialysis (PD)-associated peritonitis is a leading cause of technique failure. We aimed to develop and internally validate a Cox regression‑based nomogram for predicting peritonitis‑free survival in incident PD patients. This single-center retrospective cohort study included 372 patients who underwent peritoneal dialysis between June 2018 and December 2024, with follow-up through December 2025. LASSO‑Cox regression was used to select predictors from 23 candidate variables. A nomogram was constructed to predict 6‑, 12‑, and 24‑month peritonitis‑free survival. Model performance was assessed by concordance index (C‑index), time‑dependent ROC, calibration curves, and decision curve analysis. Over a median follow‑up of 26.3 months, 92 (35.4%) and 34 (30.4%) peritonitis events occurred in the training and validation cohorts, respectively. Nine predictors (education, start age, BMI, hemoglobin, potassium, sodium, albumin, calcium, phosphorus) were retained. Independent predictors were lower education (≤9 vs >9 years: HR 1.93, 95% CI 1.03–3.64), lower hemoglobin (HR 0.99, 0.97–1.00), lower albumin (HR 0.92, 0.87–0.97), and lower phosphorus (HR 0.53, 0.29–0.99). The C‑index was 0.707 (95% CI 0.650–0.765). Time‑dependent ROCs in the training cohort were 75.8%, 77.5%, and 69.7% at 6, 12, and 24 months; in the validation cohort, they were 77.7%, 63.8%, and 58.4%, respectively. Calibration was satisfactory at 6 and 12 months (Brier scores 6.0%–11.1%), with wider clinical net benefit at 12–24 months. This nomogram using nine routinely available variables demonstrated moderate discrimination and satisfactory calibration for predicting peritonitis‑free survival in PD patients. While promising, the model requires external validation in larger, multicenter cohorts to confirm its generalizability before routine clinical use.

## Introduction

Peritoneal dialysis (PD) is a well-established home-based kidney replacement therapy that offers patients substantial autonomy, preservation of vascular access, and quality-of-life advantages over in-center hemodialysis [1,2]. However, the long-term viability of PD as a sustained therapeutic modality is critically contingent on the prevention of infectious complications, among which PD-associated peritonitis remains the most consequential [3,4]. Peritonitis is the leading cause of technique failure, accounting for a substantial proportion of permanent transfers to hemodialysis, and is independently associated with increased mortality, prolonged hospitalization, and significant healthcare expenditure [5–7]. Despite iterative refinements in connection technology, catheter design, and prophylactic antibiotic protocols over recent decades, contemporary data from the Peritoneal Dialysis Outcomes and Practice Patterns Study (PDOPPS) indicate that crude peritonitis rates still range from 0.26 to 0.40 episodes per patient-year across different regions, underscoring the persistent and global nature of this challenge [8–10]. Approximately 10–15% of peritonitis episodes ultimately result in catheter removal and permanent hemodialysis transfer, with each additional episode further eroding patient survival [11].

The identification of patients at elevated risk for peritonitis before an episode occurs represents a clinical priority. Established risk factors include older age, diabetes mellitus, and markers of nutritional—most notably hypoalbuminemia, which consistently emerges as an independent predictor of both peritonitis occurrence and treatment failure [3,12–14]. Anemia, hypophosphatemia, and elevated inflammatory indices such as the neutrophil-to-lymphocyte ratio and C-reactive protein have been further implicated [13,15–17]. Recently, socioeconomic and behavioral determinants including educational attainment, health literacy, and patient engagement have gained recognition as modifiable factors influencing peritonitis risk through their impact on self-care capacity and adherence to aseptic technique [18–21]. Despite this substantial body of evidence, the independent contributions of these diverse risk factors have not been systematically integrated into a validated, clinically actionable prediction tool for peritonitis-free survival in PD patients.

Several prediction models have been proposed for PD-associated peritonitis, mainly using logistic regression to predict short-term outcomes, such as first onset or treatment failure. However, these studies also have some methodological issues, including small sample sizes, single-center designs, improper handling of missing data, and almost universal lack of external validation, which results in a high risk of bias. Moreover, the existing models predominantly rely on specialized biomarkers—such as dialysate neutrophil gelatinase-associated lipocalin or glucose to lymphocyte ratio—that, while biologically informative, are not routinely measured in standard clinical practice, thereby limiting their real-world applicability [17,22–24]. Critically, most of these studies have not adopted a survival analysis framework, which is the methodologically appropriate approach for modeling time-to-event outcomes such as peritonitis-free survival, nor have they systematically applied penalized regression techniques to address the high-dimensional, collinear nature of candidate predictors.

This study addresses these gaps by leveraging a longitudinal cohort of incident PD patients to: (1) identify independent predictors of peritonitis-free survival using LASSO-Cox regression; (2) construct a parsimonious nomogram integrating routine clinical variables; and (3) evaluate its discrimination, calibration, and clinical utility through internal validation, time-dependent ROC, and decision curve analysis.

## Materials and methods

This single-center, retrospective cohort study was conducted at the Fifth Affiliated Hospital of Sun Yat-sen University. Consecutive patients who initiated peritoneal dialysis (PD) *via* first catheter insertion between June 1, 2018 and December 31, 2024 were screened. Patients were followed from PD initiation until the first occurrence of peritonitis, censoring, or the end of the study period (December 31, 2025).

### Inclusion and exclusion criteria

Patients were eligible for inclusion if they met all of the following criteria: (1) aged 18 years or older at the time of PD initiation; (2) underwent first PD catheter insertion and initiated PD at our center between June 1, 2018 and December 31, 2024; (3) had complete baseline clinical data, with missingness of key predictive variables below 20%; and (4) received regular follow-up at our center, with a minimum follow-up duration of at least one year post-catheter insertion or until the occurrence of a censoring event (peritonitis, death, transfer to hemodialysis, or kidney transplantation). Patients were excluded if they met any of the following criteria: (1) had a history of peritoneal dialysis or intra-abdominal infection prior to the index catheter insertion; (2) discontinued PD within 30 days of catheter insertion for non-medical reasons; or (3) had severely incomplete follow-up data that precluded the ascertainment of the outcome status.

### Data collection

Baseline demographic and clinical data were extracted from electronic medical records. The candidate predictors (23 variables, listed in Table 1) included demographics, dialysis adequacy, and routine laboratory tests. All measurements followed standard clinical protocols.

**Table 1.** Baseline Demographic and Clinical Characteristics of the Study Cohort (*n* = 372).

Variables	Total (*n* = 372)	Training (*n* = 260)	Validation (*n* = 112)	Statistic	P
Start age, years	48.99 ± 13.26	48.24 ± 13.40	50.72 ± 12.81	t = 1.66	0.098
BMI, kg/m^2^	23.15 ± 3.28	23.35 ± 3.36	22.69 ± 3.04	t=-1.80	0.073
Weekly total Kt/V	2.09 ± 0.51	2.07 ± 0.51	2.14 ± 0.49	t = 1.19	0.235
Weekly total CrCl, L/1.73m²	75.29 ± 25.80	75.09 ± 26.71	75.78 ± 23.66	t = 0.24	0.813
Hemoglobin, g/L	108.86 ± 18.99	108.57 ± 18.41	109.54 ± 20.33	t = 0.45	0.650
Parathyroid hormone, pmol/L	26.05 ± 30.65	27.55 ± 35.17	22.57 ± 15.37	t=-1.44	0.151
25-Hydroxyvitamin D, ng/ml	12.20 ± 5.37	11.88 ± 5.05	12.94 ± 6.00	t = 1.76	0.079
Urea, mmol/L	19.42 ± 5.99	19.37 ± 5.80	19.52 ± 6.44	t = 0.22	0.822
Serum creatinine, μmol/L	849.73 ± 305.12	864.69 ± 303.08	815.00 ± 308.36	t=-1.44	0.150
Potassium, mmol/L	3.88 ± 0.66	3.86 ± 0.67	3.91 ± 0.64	t = 0.63	0.530
Sodium, mmol/L	140.61 ± 3.06	140.60 ± 3.09	140.64 ± 3.01	t = 0.13	0.894
Chloride, mmol/L	100.27 ± 4.63	100.31 ± 4.87	100.18 ± 4.05	t=-0.24	0.813
Total cholesterol, mmol/L	4.23 ± 1.90	4.30 ± 2.15	4.06 ± 1.10	t=-1.09	0.276
Low-density lipoprotein, mmol/L	2.27 ± 1.01	2.29 ± 1.05	2.21 ± 0.91	t=-0.73	0.464
Albumin, g/L	32.78 ± 4.54	32.72 ± 4.72	32.91 ± 4.09	t = 0.36	0.722
Calcium, mmol/L	2.16 ± 0.25	2.17 ± 0.28	2.15 ± 0.18	t=-0.66	0.510
Phosphorus, mmol/L	1.55 ± 0.45	1.55 ± 0.46	1.55 ± 0.43	t=-0.01	0.993
β_2_-microglobulin, mg/L	26.23 ± 11.15	26.29 ± 10.44	26.11 ± 12.69	t=-0.14	0.889
Male, n (%)	213 (57.26)	154 (59.23)	59 (52.68)	χ²=1.37	0.241
Education ≤ 9 years, n (%)	273 (73.39)	186 (71.54)	87 (77.68)	χ²=1.51	0.219
Smoker, n (%)	98 (26.34)	68 (26.15)	30 (26.79)	χ²=0.02	0.899
Diabetes, n (%)	151 (40.59)	110 (42.31)	41 (36.61)	χ²=1.05	0.304
≤3 PD exchanges per day, n (%)	151 (40.59)	102 (39.23)	49 (43.75)	χ²=0.66	0.416

Data are presented as mean ± standard deviation, median (interquartile range) or n (%).

BMI, body mass index; Weekly total CrCl, Weekly total creatinine clearance.

*p* < 0.05 indicates that the test is statistically significant.

### Outcome definition

The primary outcome of this study was the time to the first occurrence of peritoneal dialysis-associated peritonitis. The diagnosis of peritonitis was made according to the International Society for Peritoneal Dialysis (ISPD) guidelines, requiring at least two of the following three criteria: (1) clinical features consistent with peritonitis, such as abdominal pain and/or cloudy dialysis effluent; (2) dialysis effluent white blood cell count > 100/μL, with > 50% polymorphonuclear neutrophils; and (3) a positive dialysis effluent culture. Patients who did not experience peritonitis during follow-up were censored at the date of death, transfer to hemodialysis, kidney transplantation, loss to follow-up, or the end of the study period (December 31, 2025), whichever occurred first.

### Statistical analysis

No formal sample size calculation was performed *a priori*. With 92 events in training and 9 LASSO-selected predictors, the events-per-variable (EPV) was 10.2, meeting the recommended minimum [25]. The cohort was randomly split 7:3 into training (*n* = 260) and validation (*n* = 112) cohorts. Missing data were handled by single imputation: mean for normal continuous, median for skewed continuous, mode for categorical variables. We recognize that this approach may underestimate uncertainty and introduce bias. However, given the very low proportion of missing data (overall <5% for all variables), this approach was considered pragmatically acceptable, and its potential impact is addressed in the limitations.

Baseline characteristics were summarized as mean ± standard deviation for normally distributed continuous variables, median (interquartile range) for non-normally distributed continuous variables, and frequency (percentage) for categorical variables. Differences between the training and validation cohorts were compared using the Student’s t-test or Mann–Whitney U test for continuous variables and the chi-square test or Fisher’s exact test for categorical variables, as appropriate.

In the training cohort, candidate predictors were screened using univariate Cox proportional hazards regression (presented descriptively in Supplementary Table S1). To address multicollinearity and to select the most parsimonious set of predictors, the least absolute shrinkage and selection operator (LASSO) was applied using a Cox regression framework with 10-fold cross-validation. All 23 candidate variables, regardless of their univariable significance, were entered into the LASSO regression. The optimal penalty parameter (λ) was determined based on the one-standard-error rule. Variables with non-zero coefficients at this λ were retained. These selected variables were subsequently entered into a multivariable Cox proportional hazards regression model to construct the final prediction model. Hazard ratios (HRs) with 95% confidence intervals (CIs) were calculated for each predictor. A nomogram was then constructed to visualize the multivariable model and to estimate the probability of remaining peritonitis-free at 6, 12, and 24 months. The proportional hazards assumption for the final Cox model was assessed using Schoenfeld residual tests. No significant violations were detected (global test *p* > 0.05), supporting the appropriateness of the model.

The discriminative ability of the nomogram was assessed using the concordance index (C-index) and time-dependent receiver operating characteristic (ROC) curves at 6, 12, and 24 months, with the area under the ROC curve (AUC) reported. Calibration, which measures the agreement between predicted probabilities and observed event rates, was evaluated using calibration plots and quantified by the Brier score at the same time points. The clinical utility of the nomogram was assessed by decision curve analysis (DCA), which estimates the net benefit of using the model to guide risk-stratified decision-making across a range of threshold probabilities. All performance metrics were evaluated in both the training and validation cohorts (derived from internal split-sample validation).

All statistical analyses were performed using R software (version 4.4.2; R Foundation for Statistical Computing, Vienna, Austria). The following R packages were used: glmnet for LASSO regression, survival for Cox regression, rms for nomogram construction, timeROC for time-dependent ROC analysis, and ggDCA for decision curve analysis. A two-sided P value < 0.05 was considered statistically significant.

### Ethics statement

This study was approved by the Ethics Committee of the Fifth Affiliated Hospital of Sun Yat-sen University (Approval No.: ZSWY-2026-K151-1), which approved the study protocol and waived the requirement for written informed consent due to the retrospective nature of the study. The study was conducted in accordance with the principles of the Declaration of Helsinki.

## Results

### Patient characteristics

A total of 372 patients were enrolled, split into training (*n* = 260) and validation (*n* = 112) cohorts (Figure 1). Median follow-up was 26.3 months (IQR 16.5–42.2); 92 (35.4%) and 34 (30.4%) peritonitis events occurred in training and validation, respectively.

**Figure 1.**
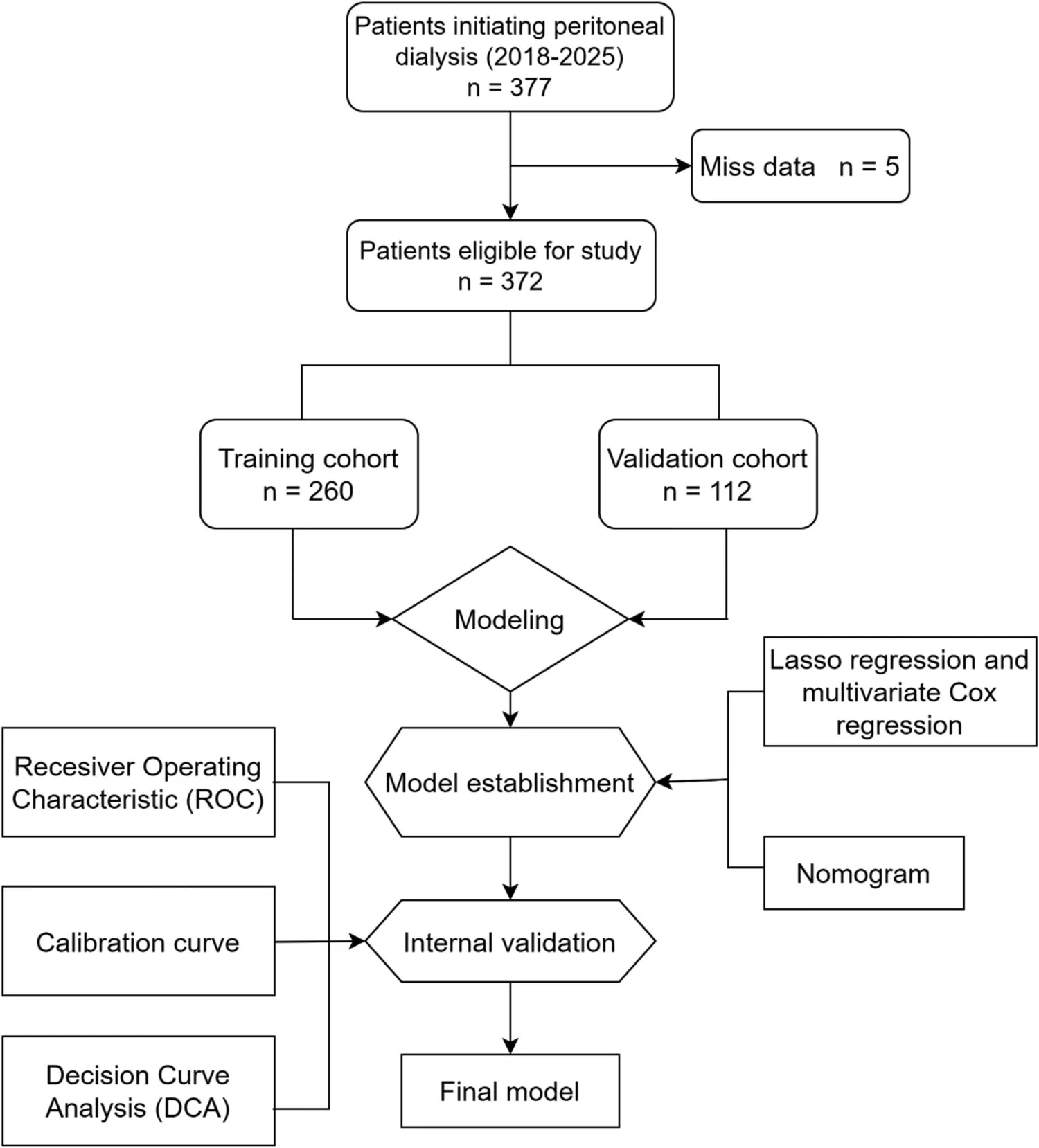
Flowchart for construction of predictive models.

The baseline demographic and laboratory characteristics of the total cohort, as well as the training and validation cohorts, are summarized in Table 1. The mean age at the initiation of dialysis was 48.99 ± 13.26 years, and 57.26% of the patients were male. The prevalence of diabetes was 40.59%, and 73.39% of the participants had an education level ≤ 9 years. The mean body mass index was 23.15 ± 3.28 kg/m^2^, and the mean dialysis adequacy (Kt/V) was 2.09 ± 0.51. Baseline laboratory values were representative of a chronic dialysis population, with a mean hemoglobin of 108.86 ± 18.99 g/L, albumin of 32.78 ± 4.54 g/L, and serum phosphorus of 1.55 ± 0.45 mmol/L. There were no statistically significant differences in any of the listed variables between the training and validation cohorts (all *p* > 0.05), confirming the homogeneity of the two groups after random partitioning.

### Feature selection and model development

In the training cohort, we first evaluated 23 candidate predictors using univariate Cox proportional hazards regression (Supplementary Table S1). To address potential multicollinearity among these variables and to select the most robust predictors, we applied LASSO regression with 10-fold cross-validation. The optimal penalty parameter (λ) was chosen as 0.0339, yielding nine predictors with non-zero coefficients: education, start age, BMI, hemoglobin, potassium, sodium, albumin, calcium, and phosphorus (Supplementary Figure S1, Supplementary Table S2).

These nine variables were then entered into a multivariable Cox regression model, the results of which are presented in Table 2. In the final model, four variables emerged as statistically significant independent predictors of peritoneal dialysis-associated peritonitis. A lower educational level (≤ 9 years vs. > 9 years) was associated with a 93% increased risk of peritonitis (HR = 1.93, 95% CI: 1.03–3.64, *p* = 0.042). Lower hemoglobin (HR = 0.99 per 1 g/L increase, 95% CI: 0.97–1.00, *p* = 0.011), lower albumin (HR = 0.92 per 1 g/L increase, 95% CI: 0.87–0.97, *p* = 0.002), and lower phosphorus (HR = 0.53 per 1 mmol/L increase, 95% CI: 0.29–0.99, *p* = 0.045) each independently conferred an elevated risk. Although start age, BMI, potassium, sodium, and calcium did not reach conventional statistical significance in the multivariable analysis (all *p* > 0.05), they were retained in the model based on their LASSO-informed selection and their incremental contribution to model performance. The overall concordance index of this model was 0.707 (95% CI: 0.650–0.765), indicating moderate discriminative ability. Assessment of Schoenfeld residuals showed no violation of the proportional hazards assumption (global *p* = 0.11) (presented descriptively in Supplementary Table S3 and Figure S4).

**Table 2.** Results of multivariate Cox regression for training cohort (*n* = 260).

Characteristic	N	Event N	HR	95% CI	P
Education					
>9 years	71	14	—	—	
0 ≤ 9 years	189	78	1.93	1.03, 3.64	0.042
Start age, years	260	92	1.01	0.99, 1.03	0.237
Body Mass Index	260	92	1.05	0.98, 1.14	0.172
Hemoglobin	260	92	0.99	0.97, 1.00	0.011
Potassium	260	92	1.33	0.93, 1.90	0.122
Sodium	260	92	0.95	0.89, 1.02	0.130
Albumin	260	92	0.92	0.87, 0.97	0.002
Calcium	260	92	0.66	0.20, 2.12	0.482
Phosphorus	260	92	0.53	0.29, 0.99	0.045

Abbreviations: CI = confidence interval, HR = hazard ratio.

For clinical applicability, we constructed a nomogram integrating these nine predictors to estimate the probability of remaining peritonitis-free at 6, 12, and 24 months (Figure 2).

**Figure 2.**
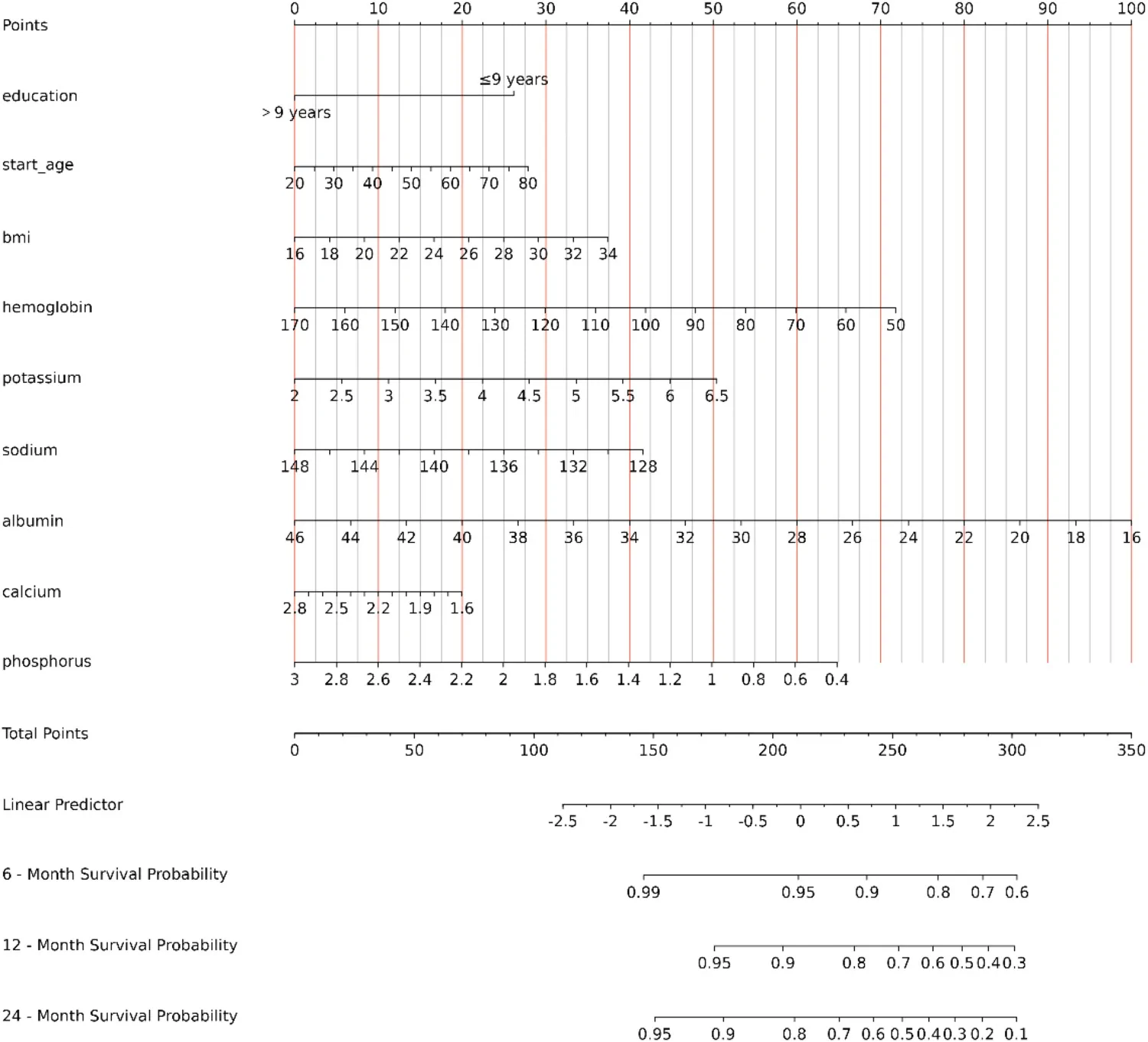
Nomogram for predicting peritonitis-free survival. The nomogram integrates nine independent predictors selected by LASSO–Cox regression. To estimate the probability of remaining peritonitis-free, locate each predictor value on its corresponding axis, draw a vertical line to the “Points” axis to obtain the assigned score, sum all scores, and read the corresponding 6-, 12-, and 24-month survival probabilities on the lowest axes.

### Model performance and validation

The discriminative performance of the nomogram was assessed using time-dependent ROC curves at 6, 12, and 24 months (Figure 3). In the training cohort, the AUC was 75.8% (95% CI: 64.4%–87.3%) at 6 months, 77.5% (95% CI: 69.3%–85.8%) at 12 months, and 69.7% (95% CI: 61.3%–78.0%) at 24 months. The model demonstrated the highest discriminative ability at 12 months post-initiation of peritoneal dialysis. In the validation cohort, the corresponding AUC values were 77.7% (95% CI: 66.8%–88.6%) at 6 months, 63.8% (95% CI: 47.4%–80.1%) at 12 months, and 58.4% (95% CI: 45.4%–71.4%) at 24 months.

**Figure 3.**
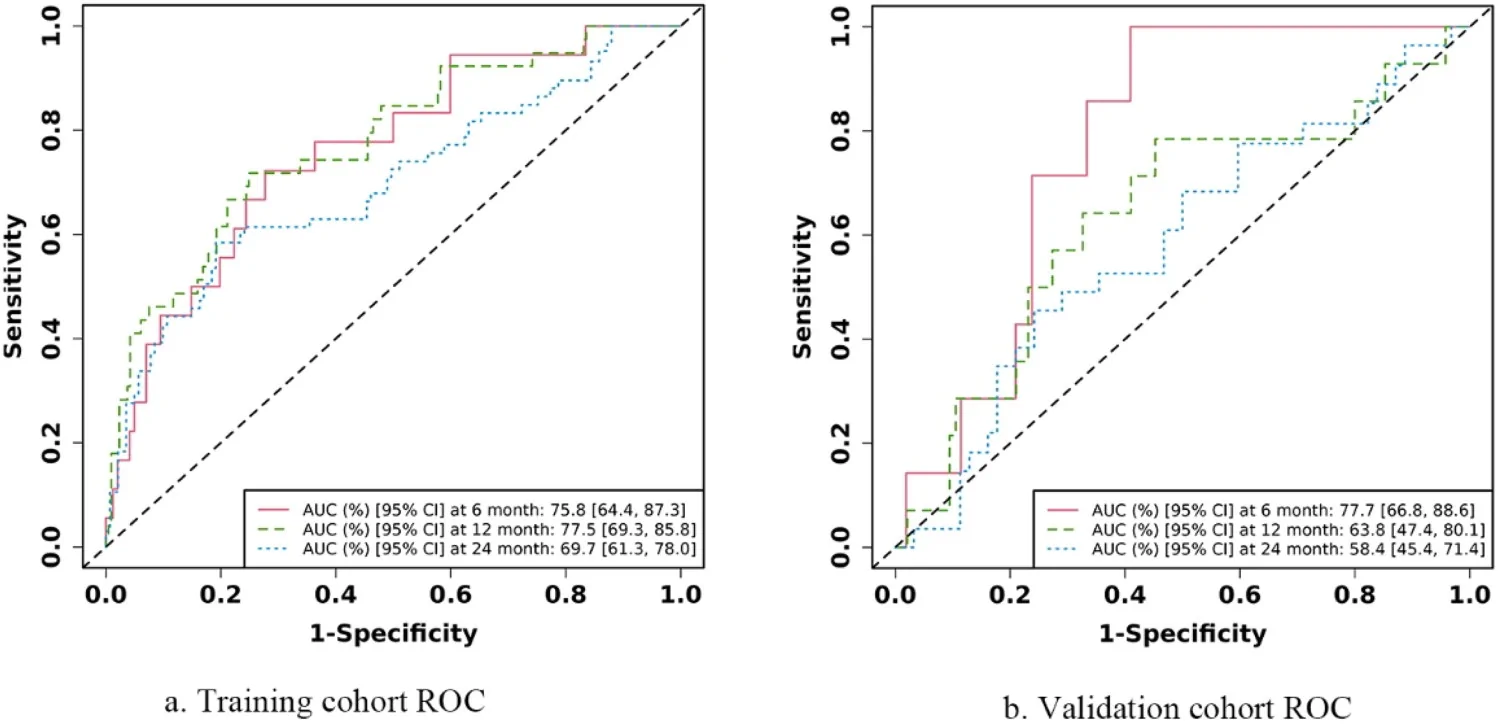
Time-dependent receiver operating characteristic (ROC) curves of the nomogram.

Calibration of the nomogram was assessed at 6, 12, and 24 months in both the training and validation cohorts (Figure 4). Calibration curves plot the predicted probability against the actual observed event rate, with closer alignment to the 45-degree diagonal indicating better calibration. In the training cohort, the nomogram demonstrated excellent calibration across all three time points. At 6 months, the calibration curve closely followed the ideal line, yielding a low Brier score of 6.0% (95% CI: 3.6%–8.3%). Good calibration was maintained at 12 months (Brier score = 10.7%, 95% CI: 8.1%–13.4%) and 24 months (Brier score = 16.7%, 95% CI: 13.9%–19.5%), with only minor deviations at the higher range of predicted probabilities. In the validation cohort, the nomogram retained satisfactory calibration at 6 months, with a Brier score of 5.6% (95% CI: 2.0%–9.2%), comparable to the training cohort performance. At 12 months, the calibration curve remained reasonably close to the ideal line, although a slight overestimation of risk was observed (Brier score = 11.1%, 95% CI: 6.9%–15.2%). At 24 months, the model exhibited a more noticeable overestimation of peritonitis risk, particularly for patients with predicted probabilities in the middle-to-high range; this is reflected in the higher Brier score of 19.9% (95% CI: 15.3%–24.5%). The gradual increase in Brier scores from 6 to 24 months is expected, as longer prediction horizons inherently carry greater uncertainty.

**Figure 4.**
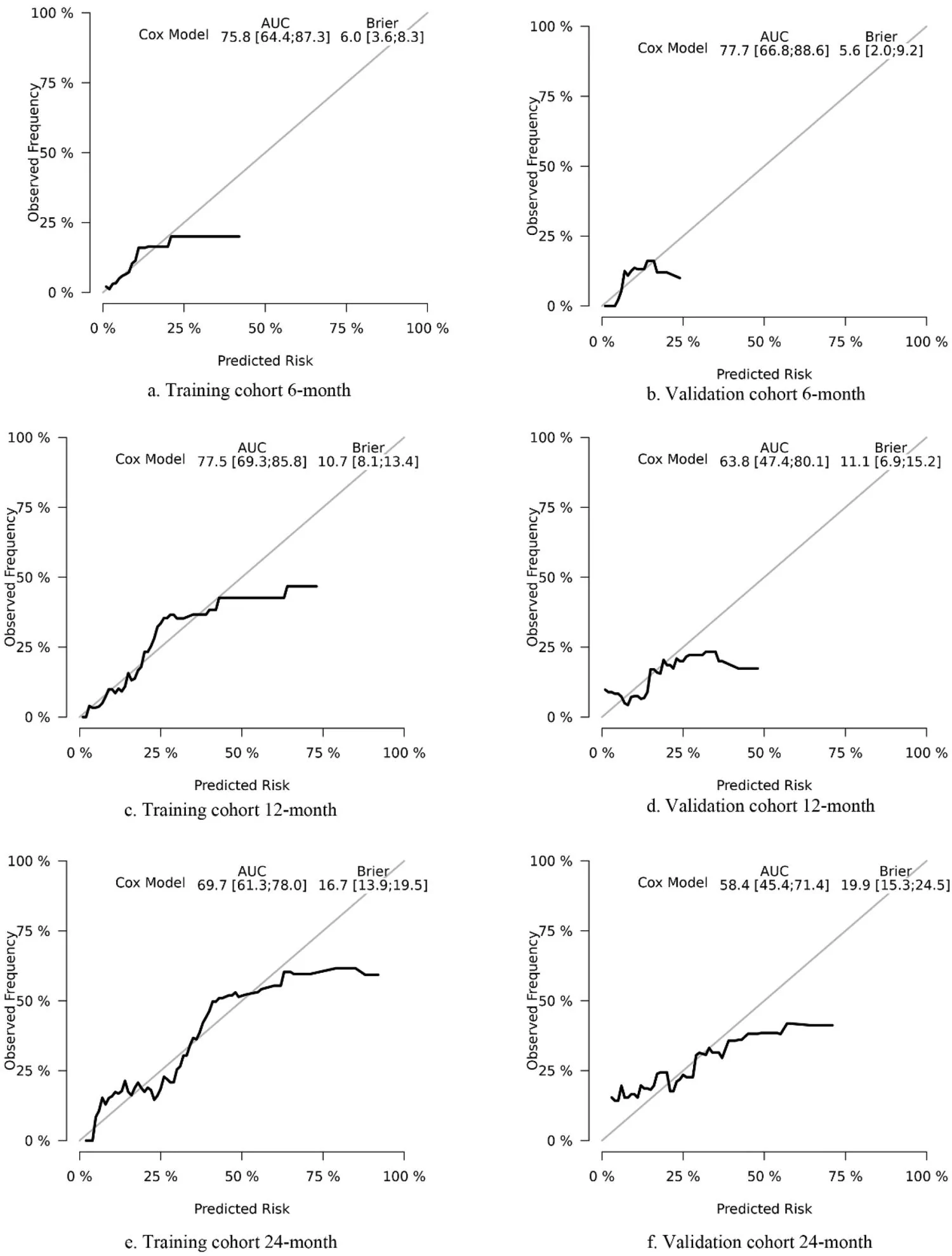
Calibration curves of the nomogram at 6-, 12-, and 24-month.

The clinical utility of the nomogram was evaluated using decision curve analysis across 6-, 12-, and 24-month time points in both the training and validation cohorts (Figure 5). For the 6-month prediction model, the net benefit was limited to a narrow range of threshold probabilities—0 to 20% in the training cohort and 0 to 15% in the validation cohort—indicating marginal clinical applicability at this early time point. For the 12-month model, a substantially broader clinical utility was observed, with net benefit exceeding both reference strategies across threshold probabilities of 0 to 65% in the training cohort (peak net benefit ≈ 0.15); in the validation cohort, the advantage was maintained within the clinically relevant range of 0 to 20%. For the 24-month model, the nomogram achieved the widest clinical applicability, demonstrating net benefit across 0 to 90% of threshold probabilities in the training cohort (peak net benefit > 0.2) and 0 to 30% in the validation cohort. Collectively, these results indicate that the nomogram conferred meaningful net clinical benefit at 12 and 24 months, with the 24-month model exhibiting the greatest potential to inform risk-stratified clinical decision-making.

**Figure 5.**
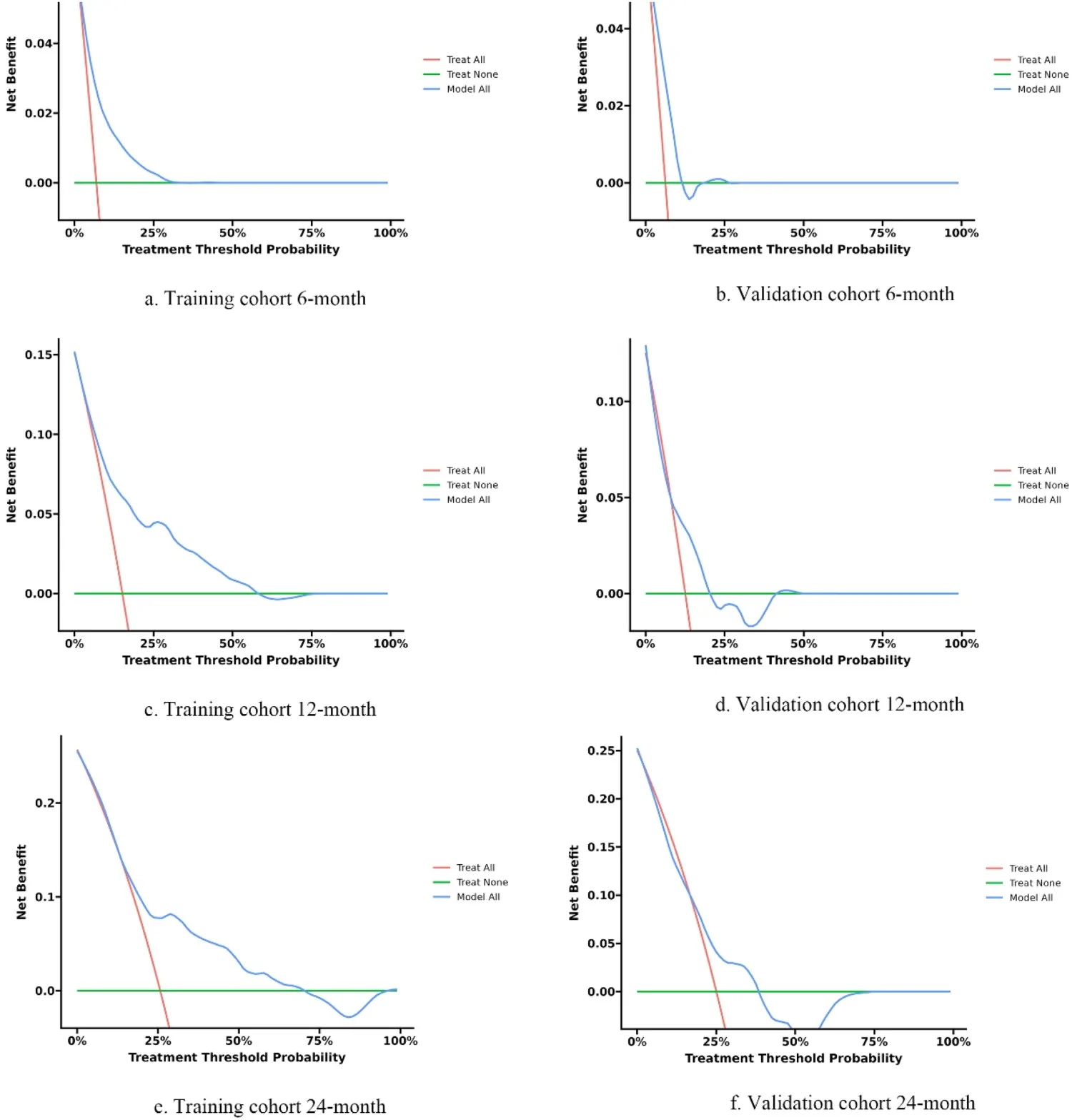
Decision curve analysis (DCA) of the nomogram at 6-, 12-, and 24- months.

### Sensitivity analysis

To justify the retention of all nine LASSO-selected predictors, we constructed a reduced model containing only the four statistically significant variables (education, hemoglobin, albumin, and phosphorus) and compared its performance with the full 9-predictor model in the training cohort. The full model demonstrated superior discriminative ability, with a higher C-index (0.707 vs. 0.660 for the reduced model). Time-dependent AUCs of the complete model remained consistently higher than those of the simplified model at both 12 months and 24 months (Supplementary Figure S2). Calibration analysis further revealed that the reduced model showed greater overestimation of risk, particularly at the 12‑ and 24‑month time points (Supplementary Figure S3). These findings support our decision to retain all LASSO‑selected variables in the final model, as the full model provided better discrimination and calibration compared with the more parsimonious alternative.

## Discussion

In this single-center, retrospective cohort study, we developed and internally validated a Cox regression-based nomogram for predicting the first occurrence of peritoneal dialysis-associated peritonitis. Leveraging LASSO regression for variable selection from 23 candidate predictors, the final model incorporated nine variables—education, start age, BMI, hemoglobin, potassium, sodium, albumin, calcium, and phosphorus—among which lower educational attainment, lower hemoglobin, lower albumin, and lower phosphorus emerged as statistically significant independent risk factors. The nomogram demonstrated moderate discriminative ability (C-index = 0.707) and satisfactory calibration, with the greatest clinical utility observed for predicting peritonitis risk at 12 and 24 months. The observed decline in performance metrics from the training to the validation cohort, particularly the AUC at 24 months, is noteworthy. While partly attributable to the smaller validation sample and the inherent difficulty in long-term prediction, this attenuation may also indicate some degree of model optimism or overfitting, which is a common challenge in single-center prediction model studies.

The identification of lower educational attainment (≤ 9 years) as an independent predictor of peritonitis merits particular attention. In our study, this association was of marginal statistical significance (HR = 1.93, 95% CI: 1.03–3.64, *p* = 0.042), which suggests a potential role for educational level in influencing peritonitis risk, but this finding should be interpreted cautiously. While the relationship between health literacy and PD outcomes has been increasingly recognized, education has seldom been formally integrated into predictive models for peritonitis [2,18]. Our finding extends this literature by showing that educational level—a fundamental determinant of health literacy—may directly influence a patient’s ability to maintain sterile technique, recognize early signs of infection, and communicate effectively with healthcare providers [26,27]. Importantly, assistance in PD exchange procedures has been shown to mitigate peritonitis risk related to breaches in aseptic technique [28]. This suggests that patients with lower educational attainment, who may face greater challenges in mastering the procedural complexity of PD, could benefit substantially from targeted training programs or assisted PD.

The significant associations of lower hemoglobin, albumin, and phosphorus with peritonitis risk are biologically plausible and consistent with the established literature on the malnutrition-inflammation complex syndrome (MICS) in dialysis patients [15,16,29]. Hypoalbuminemia is one of the most consistent predictors of peritonitis across multiple cohorts, reflecting both protein-energy wasting and chronic inflammation [3]. In our study, each 1 g/L decrease in serum albumin was associated with an 8% increase in peritonitis risk, underscoring the importance of nutritional assessment at PD initiation. The protective effect of higher hemoglobin (HR = 0.99 per 1 g/L increase, *p* = 0.011) aligns with evidence linking anemia to impaired immune function in chronic kidney disease, though it remains difficult to disentangle whether hemoglobin serves as a direct mediator or a surrogate for overall nutritional and inflammatory status [13,30]. Lower serum phosphorus was also independently associated with increased peritonitis risk (HR = 0.53, *p* = 0.045). Hypophosphatemia in PD patients frequently indicates poor dietary intake and protein-energy wasting, and has previously been linked to increased mortality in dialysis populations [31,32]. Collectively, these three laboratory parameters—albumin, hemoglobin, and phosphorus—paint a coherent picture of nutritional vulnerability that predisposes patients to infectious complications. Their inclusion in the nomogram provides a quantitative framework for translating these well-known risk factors into individualized risk estimates.

The performance of our nomogram indicates moderate discriminative ability that is consistent with or slightly lower than existing prediction models for peritonitis. For instance, Yan et al. developed a nomogram for refractory peritonitis with an AUC of 0.781 in their training cohort [14], while Wang et al. reported an AUC of 0.929 for predicting any peritonitis episode [13]. Direct comparisons are challenging due to differences in outcome definitions (e.g. first episode vs. refractory peritonitis), follow-up durations, and predictor sets. Many previous models also relied on specialized biomarkers not routinely available, limiting their clinical applicability. The AUCs in the training cohort (75.8% at 6 months, 77.5% at 12 months, 69.7% at 24 months) suggest that the model performs best for short- to medium-term prediction, which is clinically relevant for guiding surveillance intensity and preventive interventions early in the course of PD.

Beyond predictive performance, several aspects of our study distinguish it from prior work and enhance its clinical relevance. First, unlike most existing models that rely on logistic regression for binary outcome prediction [13,14,23], we adopted a Cox proportional hazards framework with LASSO penalization, which appropriately handles time-to-event data and mitigates overfitting in the presence of multiple collinear predictors. Second, our model is built exclusively on routinely available demographic and laboratory parameters, avoiding specialized biomarkers (e.g. dialysate NGAL, glucose-to-lymphocyte ratio) that are not universally accessible in real-world clinical settings [17,22,24]. This design choice prioritizes practical utility and scalability, particularly in resource-limited environments. Third, we explicitly incorporated educational attainment as a predictor—a factor seldom integrated into previous peritonitis risk models [18,21]—reflecting the growing recognition of socioeconomic and behavioral determinants in PD outcomes. Fourth, our model provides time-to-event predictions at multiple clinically relevant time points (6, 12, and 24 months), whereas most prior models offer only static risk estimates [13,14]. While our model’s discriminative ability is moderate compared with some biomarker-enriched models, its accessibility and ease of application may facilitate broader adoption for initial risk stratification at PD initiation. Future iterations could incorporate additional predictors (e.g. health literacy assessments, serial nutritional markers) to further enhance performance.

The calibration of the nomogram was excellent in the training cohort and satisfactory in the validation cohort, particularly at 6 and 12 months. Brier scores increased from approximately 6% at 6 months to 17%–20% at 24 months, reflecting the inherent difficulty of predicting events over longer horizons—a pattern commonly observed in survival prediction models. The modest attenuation of discrimination and calibration in the validation cohort, including lower AUCs (63.8% at 12 months, 58.4% at 24 months), highlights the need for external validation in larger, multicenter cohorts before the nomogram can be recommended for widespread clinical use. It is also important to note that the final model retained nine predictors, of which only four were statistically significant in the multivariable Cox regression. This retention was based on their LASSO-informed selection, which can improve model stability and predictive performance by accounting for the joint effects of all variables. To further justify this, we conducted a sensitivity analysis comparing the full 9-predictor model with a more parsimonious model containing only the four significant predictors. The full model showed a slightly higher C-index (0.707 vs. 0.660) and improved calibration, particularly at 12 and 24 months, supporting our decision to retain all LASSO-selected variables.

Decision curve analysis provided additional insights into the clinical utility of the nomogram. The 6-month model showed limited net benefit, only surpassing the reference strategies within a narrow threshold probability range (0–20% in training, 0–15% in validation). In contrast, the 12- and 24-month models demonstrated substantially broader clinical applicability, with the 24-month model achieving the widest threshold range in the training cohort (0–90%, peak net benefit > 0.2). These findings suggest that the nomogram is most clinically useful for predicting medium- to long-term peritonitis risk, where the window for preventive intervention is wider and risk-stratified approaches may yield greater absolute benefit.

Several limitations of this study should be carefully considered. First, the single-center, retrospective design introduces potential selection bias and limits generalizability. The model’s performance, while acceptable, showed some decline upon validation, and the wide confidence intervals around the AUC estimates in the validation cohort indicate limited statistical power. This suggests that the model may be overly optimistic, and the reported performance metrics are likely to be lower when applied to new populations. Second, while the proportion of missing data was low, our use of single imputation rather than multiple imputation may underestimate uncertainty and introduce bias. Third, some established predictors of peritonitis were not included in the study, which had to be abandoned based on the actual situation of the center, the time point at which the prediction model was established, and the sample size. Fourth, death and transfers were treated as censoring rather than competing risks; however, the bias is likely small given the young cohort (mean age 49 years) and low 2-year mortality (<5%). Fifth, and most importantly, this study utilized a random split-sample internal validation. While this is an acceptable first step, it does not substitute for true external validation in an independent, ideally multicenter, cohort. Therefore, our nomogram should be viewed as a promising preliminary tool requiring further validation before it can be recommended for routine clinical decision-making.

## Conclusion

In summary, we developed and internally validated a nomogram integrating nine routinely available clinical variables to predict peritonitis-free survival in patients initiating peritoneal dialysis. Lower educational attainment, lower hemoglobin, lower albumin, and lower phosphorus were identified as independent risk factors. The nomogram demonstrated moderate discrimination, satisfactory calibration, and meaningful clinical net benefit at 12 and 24 months. This preliminary tool may assist clinicians in identifying high-risk patients at the initiation of PD, potentially enabling early risk-stratified preventive interventions. However, external validation in larger, diverse populations is essential before its widespread adoption.

## Supplementary Material

Supplemental Material

## Data Availability

The datasets used and/or analyzed during the current study are available from the corresponding author on reasonable request.
