# Supplementary material for: Development and validation of a nomogram for peritoneal dialysis-associated peritonitis
Source: Ren Fail. 2026 Oct 1;48(1):2724570. doi: 10.1080/0886022X.2026.2724570 (PMC13637786; doi:10.1080/0886022X.2026.2724570)
Supplement: Supplementary file 1 [file IRNF_A_2724570_SM5460.docx]

Table S1 Results of Univariate Cox regression for Training Cohort (n = 260)

| **Variables** | **β** | **S.E** | **Z** | **P** | **HR (95%CI)** |
| --- | --- | --- | --- | --- | --- |
| Sex |  |  |  |  |  |
| male |  |  |  |  | 1.00 (Reference) |
| female | 0.11 | 0.21 | 0.51 | 0.611 | 1.11 (0.74, 1.68) |
| Education |  |  |  |  |  |
| ＞9 years |  |  |  |  | 1.00 (Reference) |
| ≤9 years | -0.72 | 0.28 | -2.53 | 0.011 | 0.49 (0.28, 0.85) |
| Smoker |  |  |  |  |  |
| no |  |  |  |  | 1.00 (Reference) |
| yes | -0.16 | 0.24 | -0.63 | 0.526 | 0.86 (0.53, 1.38) |
| Diabetes |  |  |  |  |  |
| no |  |  |  |  | 1.00 (Reference) |
| yes | 0.54 | 0.21 | 2.56 | 0.011 | 1.72 (1.13, 2.60) |
| PD exchanges per day |  |  |  |  |  |
| ＞3 |  |  |  |  | 1.00 (Reference) |
| ≤3 | -0.21 | 0.21 | -1.00 | 0.319 | 0.81 (0.54, 1.22) |
| Start age | 0.04 | 0.01 | 4.71 | <.001 | 1.04 (1.02, 1.05) |
| BMI | 0.06 | 0.03 | 1.90 | 0.057 | 1.06 (1.00, 1.12) |
| Weekly total Kt/V | -0.16 | 0.21 | -0.77 | 0.442 | 0.85 (0.56, 1.28) |
| Weekly total CrCl | 0.00 | 0.00 | 0.62 | 0.537 | 1.00 (1.00, 1.01) |
| Hemoglobin | -0.01 | 0.01 | -2.39 | 0.017 | 0.99 (0.98, 0.99) |
| Parathyroid hormone | -0.00 | 0.00 | -0.45 | 0.650 | 1.00 (0.99, 1.01) |
| 25-Hydroxyvitamin D | -0.02 | 0.02 | -0.95 | 0.342 | 0.98 (0.94, 1.02) |
| Urea | -0.01 | 0.02 | -0.55 | 0.580 | 0.99 (0.95, 1.03) |
| Serum creatinine | -0.01 | 0.00 | -3.14 | 0.002 | 0.99 (0.99, 0.99) |
| Potassium | 0.01 | 0.15 | 0.07 | 0.943 | 1.01 (0.75, 1.36) |
| Sodium | -0.03 | 0.03 | -1.00 | 0.316 | 0.97 (0.90, 1.03) |
| Chloride | 0.01 | 0.02 | 0.53 | 0.599 | 1.01 (0.97, 1.06) |
| Total cholesterol | 0.07 | 0.04 | 1.69 | 0.090 | 1.07 (0.99, 1.15) |
| Low-density lipoprotein | 0.11 | 0.09 | 1.16 | 0.244 | 1.12 (0.93, 1.34) |
| Albumin | -0.08 | 0.02 | -3.41 | <.001 | 0.93 (0.89, 0.97) |
| Calcium | -0.41 | 0.47 | -0.87 | 0.384 | 0.66 (0.26, 1.67) |
| Phosphorus | -0.67 | 0.25 | -2.67 | 0.008 | 0.51 (0.31, 0.84) |
| β_2_-microglobulin | -0.01 | 0.01 | -0.74 | 0.462 | 0.99 (0.97, 1.01) |

BMI, body mass index; Weekly total CrCl, Weekly total creatinine clearance.

Table S2 The coefficients of Lasso regression analysis

| Variable | Coefficient |
| --- | --- |
| sex_male | 0.000000000 |
| education_≤9 years | 0.341874193 |
| smoker_yes | 0.000000000 |
| diabetes_yes | 0.000000000 |
| start_age | 0.009986627 |
| pd_exchanges_per_day _＞3 | 0.000000000 |
| bmi | 0.015587542 |
| weekly_total_ktv | 0.000000000 |
| weekly_total_crcl | 0.000000000 |
| hemoglobin | -0.009749871 |
| parathyroid_hormone | 0.000000000 |
| 25_hydroxyvitamin_d | 0.000000000 |
| urea | 0.000000000 |
| serum_creatinine | 0.000000000 |
| potassium | 0.053010470 |
| sodium | -0.025823868 |
| chloride | 0.000000000 |
| total_cholesterol | 0.000000000 |
| low_density_lipoprotein | 0.000000000 |
| albumin | -0.069211628 |
| calcium | -0.147068685 |
| phosphorus | -0.226818863 |
| beta2_microglobulin | 0.000000000 |
